# Anti-leukemia activity of semi-synthetic phenolic derivatives from *Polygonum limbatum* Meisn.

**DOI:** 10.1186/s13065-015-0115-2

**Published:** 2015-06-24

**Authors:** Antoine Honoré Lonfouo Nkuété, Victor Kuete, Davide Gozzini, Ludovico Migliolo, Aline Lima Oliveira, Hippolyte K Wabo, Pierre Tane, Giovanni Vidari, Thomas Efferth, Octávio Luiz Franco

**Affiliations:** Department of Chemistry, Faculty of Science, University of Dschang, Dschang, Cameroon; Centro de Analises Proteômicas e Bioquimicas, Pós-Graduação em Ciencias Genomicas e Biotecnologia, Universidade Catolica de Brasilia, Brasilia, DF Brazil; Dipartimento di Chimica, Laboratorio di Chimica delle Sostanze Organiche Naturali e Centro di Etnobiofarmacia (CISTRE), Università degli Studi di Pavia, Via Taramelli, 12-27100 Pavia, Italy; Department of Pharmaceutical Biology, Institute of Pharmacy and Biochemistry, University of Mainz, Staudinger Weg 5, 55128 Mainz, Germany; Instituto de Química, Universidade de Brasília, Brasilia, DF Brazil; Department of Biochemistry, Faculty of Science, University of Dschang, Dschang, Cameroon; S-Inova Biotech, Universidade Catolica Dom Bosco, Campo Grande, MS, Brazil

**Keywords:** Phenolic compounds, Sargisin, Metapchromone, LimbachalconeA, Tsedengchalcone

## Abstract

**Background:**

The present report describes the semi-synthesis of a few O-prenylated phenolic derivatives and their in vitro antitumor activities. These compounds were prepared by modifying two naturally occurring antitumor phenols, 5,7-dihydroxy-3-(1′-hydroxy-1′-phenyl-methyl)-6-methoxy-chroman-4-one (**A**) and 2′,4′-dihydroxy-3′,6′-dimethoxychalcone (**B**), previously isolated from *Polygonum limbatum* Meisn. (Polygonaceae). The structures were elucidated by spectroscopic means and comparison with published data. The cytotoxicity of compounds was determined by using the resazurin assay in the parental drug-sensitive CCRF-CEM cell line and its multidrug-resistant P-glycoprotein-over-expressing subline, CEM/ADR5000.

**Results:**

We describe in the present paper four new semi-synthetic derivatives of **A** and **B**: 5-hydroxy-6-methoxy-7-*O*-(3′-methylbut-2′-enyl)chroman-4-one (**1**), trivially named metapchromone, 5-acetoxy-6-methoxy-7-*O*-[3′-methylbut-2′enyl]chroman-4-one (**2**), trivially named sargisin, 2′-hydroxy-3′,6′-dimethoxy-4′-*O*-(3″-methylbut-2″-enyl)chalcone (**3**) trivially named limbachalcone A, and 2′-acetoxy-3′,6′-dimethoxy-4′-*O*-(3″-methylbut-2″-enyl)chalcone (**4**) trivially named tsedengchalcone. Their preliminary cytotoxic activities have been determined. We also report herein the isolation of 1-methylhydantoin (**C**) and betulinic acid (**D**) from *Polygonum limbatum* for the first time.

**Conclusions:**

The study clearly suggests that semi-synthesis involving O-prenylation and acetylation of chalcones or other chromanones should be avoided in a search for anticancer drugs. This conclusion should be helpful when selecting substituents for the synthesis of potential anticancer drugs.

## Background

The genus *Polygonum* belongs to the family Polygonaceae, comprising 300 species growing all over the world, although most of them are found in temperate and tropical regions [1, 2]. *Polygonum limbatum* Meisn. (Polygonaceae) commonly known as *“nzùnh m*Ɛ*tap”* in the Western Region of Cameroon, is a perennial herb that grows widely in marshy and aquatic areas, near riverbanks [2]. In connection with our ongoing search for bioactive compounds, the phytochemical re-examination of *Polygonum limbatum* Meisn. allowed us to isolate 1-methylhydantoin (**C**), 5,7-dihydroxy-3-(1′-hydroxy-1′-phenyl-methyl)-6-methoxy-chroman-4-one (**A**), 2′,4′-dihydroxy-3′,6′-dimethoxychalcone (**B**), betulinic acid (**D**) and sitosterol 3-*O*-β-D-glucopyranoside (**E**). Compounds **A** and **B** were previously identified as antitumor phenolic compounds [3, 4]. In addition, in the present paper we describe four new semi-synthetic derivatives of compounds **A** and **B**: 5-hydroxy-6-methoxy-7-*O*-(3′-methylbut-2′-enyl)chroman-4-one (**1**) trivially named metapchromone, 5-acetoxy-6-methoxy-7-*O*-[3′-methylbut-2′enyl]chroman-4-one (**2**) trivially named sargisin, 2′-hydroxy-3′,6′-dimethoxy-4′-*O*-(3″-methylbut-2″-enyl)chalcone (**3**) trivially named limbachalcone A, and 2′-acetoxy-3′,6′-dimethoxy-4′-*O*-(3″-methylbut-2″-enyl)chalcone (**4**), trivially named tsedengchalcone. Their preliminary antitumor activities have been determined. We also report herein the isolation of 1-methylhydantoin (**C**) as a natural product from the *Polygonum* genus for the first time.

The isolation of 1-methylhydantoin and betulinic acid from the *Polygonum* genus and the Polygonaceae family could be an important chemotaxonomic finding.

## Results

The structures of natural compounds **A**, **B** and **C** isolated from *P. limbatum* were elucidated on the basis of spectroscopic data such as IR, 1D and 2D NMR spectra. Comparison of the data with those reported in the literature led to the identification of compounds such as betulinic acid (**D**) [5] and sitosterol 3-*O*-β-D-glucopyranoside (**E**) [6].

**Fig. 1:**
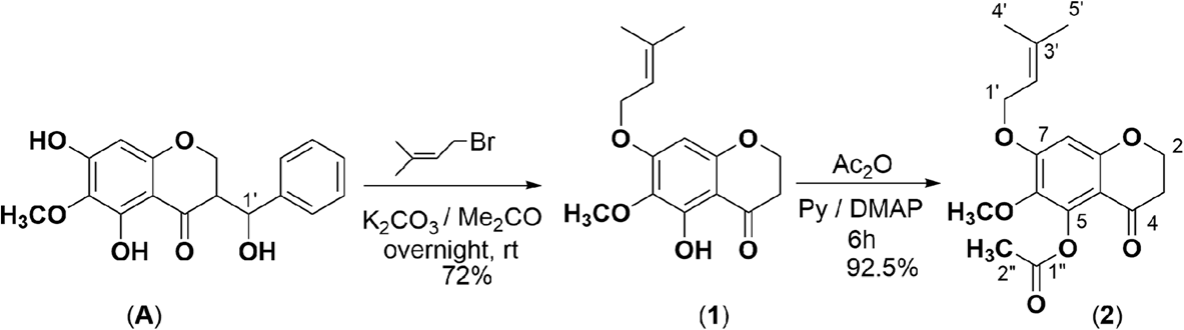
Semi-synthesis of compounds**1** and **2** from 5,7-dihydroxy-3-(1′-hydroxy-1′-phenyl-methyl)-6-methoxy-chroman-4-one (**A**)

The structures of the semi-synthetic derivatives, 5-hydroxy-6-methoxy-7-*O*-(3′-methylbut-2′-enyl)chroman-4-one (**1**), 5-acetyl-6-methoxy-7-*O*-(3′-methylbut-2′-enyl)chroman-4-one (**2**), 2′-hydroxy-3′,6′-dimethoxy-4′-*O*-(3″-methylbut-2″-enyl)chalcone (**3**), 2′-acetyl-3′,6′-dimethoxy-4′-*O*-(3″-methylbut-2″-enyl)chalcone (**4**) (Fig. 1) were determined on the basis of ^1^H NMR, ^13^C NMR and EIMS data and comparison with those of 5,7-dihydroxy-3-(1′-hydroxy-1′-phenyl-methyl)-6-methoxy-chroman-4-one (**A**), 2′,4′-dihydroxy-3′,6′-dimethoxychalcone (**B**), respectively. This is the first report concerning the isolation of 1-methylhydantoin (**C**) from *P. limbatum* as well as the semi-synthesis of prenylated and acetylated derivatives from compounds **A** and **B**.

**Fig. 2:**
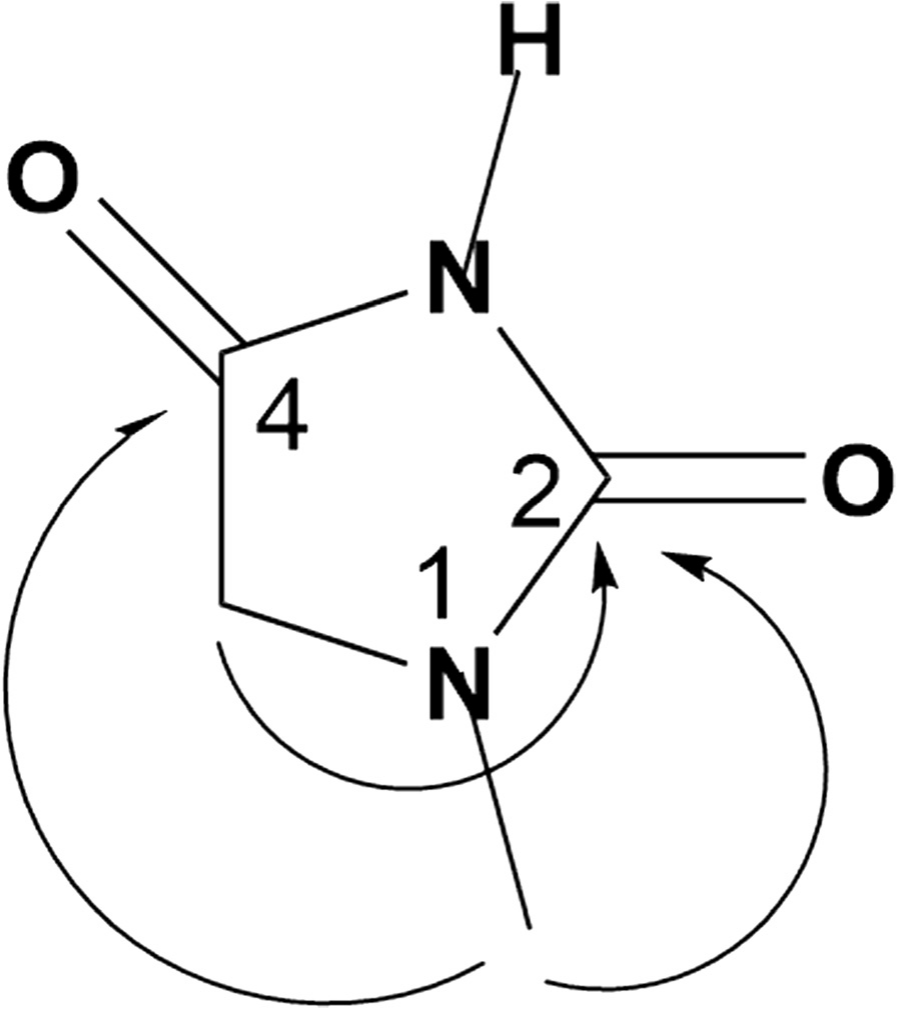
Key HMBC correlations for compound **C**

Compound **C** was obtained as brownish needles from *n*-hexane-EtOAc, mp 155–157 °C. It reacted negatively to the FeCl_3_, suggesting the absence of a phenolic group in the molecule. Its molecular formula of C_4_H_6_N_2_O_2_, corresponding to 3 degrees of unsaturation, was determined by EI-MS (M^+^*m/z* 114) in conjunction with the NMR spectra. In the ^13^C NMR spectrum, signals at *δ*_C_ 173.8 (C-4) and 159.9 (C-2) for two carbonyl groups, one methylene signal at *δ*_C_ 29.2 (C-5) and one methyl at *δ*_C_ 53.9 (N-CH_3_), were characteristic of 1-methylhydantoin structure [7]. In the ^1^H NMR spectrum, two singlets were observed at *δ*_H_ 3.95 and 2.90, respectively, assignable to these methyl and methylene groups, respectively (Table 1). In the HMBC spectrum, pertinent correlations were observed between H-5 and C-2, CH_3_ and C-2 and C-4. The structure of compound **C** was established as 1-methyldiazolidine-2,4-dione (Fig. 2). It is a natural product from the *Polygonum* genus and has been fully characterized here for the first time. It has been previously reported as a synthetic compound and was found to be a renal metabolite of dupracetam [7].

**Table 1:** Comparative NMR data of 1-Methylhydantoin

Position	Compound C 1-Methylhydantoin		Reference data (7)
	1H (MeoD) (75 MHz)	13C (MeoD) (75 MHz)	1H DMSO (100 MHz)
-CH3	2.91(s)	53.9	2.80(s)
-CH2	3.95(s)	29.2	3.86(s)
-NH-	-	-	10(br s)
C_2_=O	-	159.9	-
C_4_=O	-	173.8	-

Compounds **A** and **B** were prenylated under standard basic conditions by exposure to prenyl bromide. As expected, the poorly reactive chelated phenolic groups were not alkylated. In fact, subsequent acetylation of these groups required forcing conditions with the aid of 4-dimethylaminopyridine (DMAP) in a catalytic amount.

**Fig. 3:**
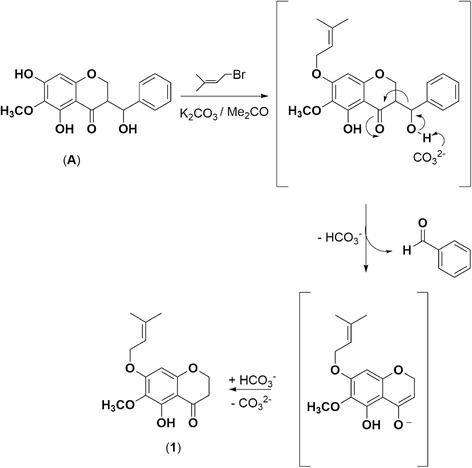
Proposed retro-aldol-like reaction for the genesis of compound **1** from **A** [12]

Interestingly, the prenylation of chroman-4-one **A** resulted in decomposition of the molecule with loss of benzaldehyde and formation of a 3-unsubstituted chromanone, namely compound **1**. This rearrangement was likely due to a retro-aldol-like reaction following the mechanism shown in Fig. 3.

## Discussion

**Fig. 4:**
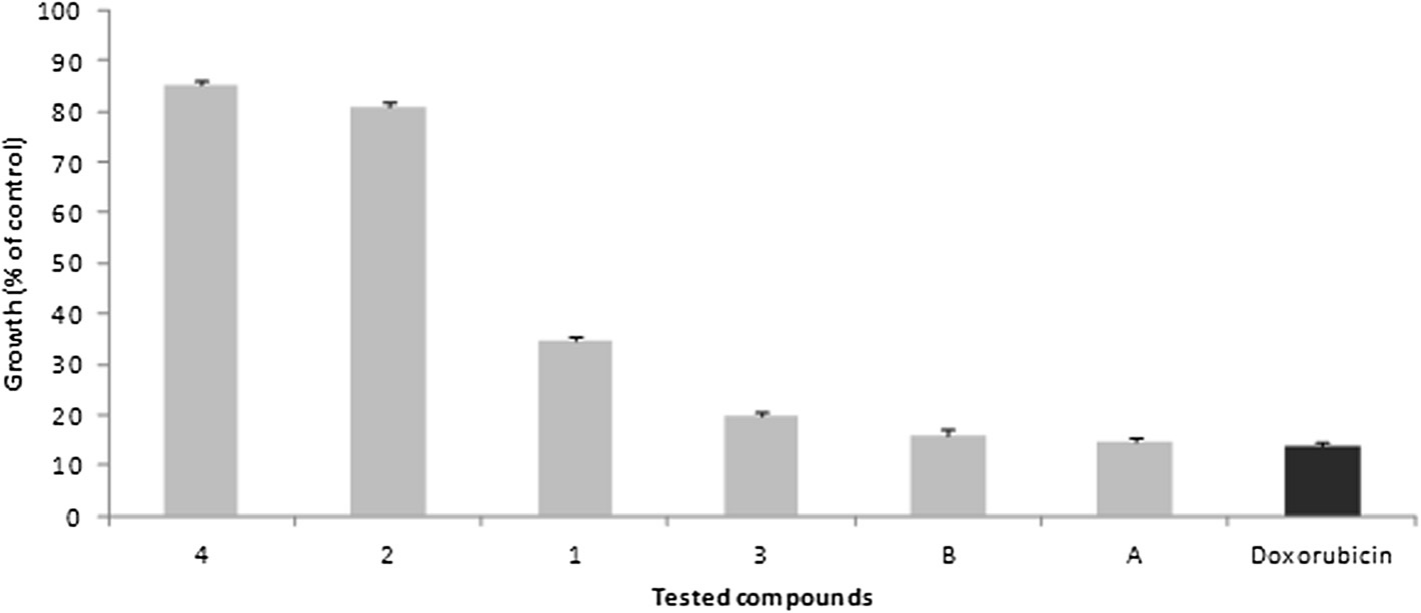
Cytotoxicity activity of compounds on leukaemia CCRF-CEM cells at 125 μM.5,7-dihydroxy-3-(1′-hydroxy-1′-phenyl-methyl)-6-methoxy-chroman-4-one (**A**), 2′,4′-dihydroxy-3′,6′-dimethoxychalcone (**B**); 5-hydroxy-6-methoxy-7-*O*-(3′,3′-dimethylprop-2′-enyl)chroman-4-one (**1**), 5-acetyl-6-methoxy-7-*O*-(3′,3′-dimethylprop-2′-enyl)chroman-4-one (**2**), 2′-hydroxy-3′,6′-dimethoxy-4′-*O*-(3″,3″-dimethylprop-2″-enyl)chalcone (**3**), 2′-acetyl-3′,6′-dimethoxy-4′-*O*-(3″,3″-dimethylprop-2″-enyl)chalcone (**4**). Doxorubicin was used as a positive control

In this study, we determined the cytotoxicity of the natural compound **A** as well as the semi-synthetic compounds **1**–**4**. We previously reported the cytotoxicity of compound **B** [3, 4], and the data were also reported herein for a better understanding of the structure-activity relationship (SAR). As shown in Fig. 4, the two natural compounds **A** and **B** were much more active than the semi-synthetic ones, inducing less than 20 % growth of CCRF-CEM leukaemia cells. This was confirmed in the dose–response assays, as IC_50_ values below 20 μM were recorded for the two natural compounds. Among the synthetic compounds (Table 2), **3** displayed the highest activity with IC_50_ values below 20 μM on the two tested leukaemia cell lines. Interestingly, the resistant cell line was more sensitive to compound **3** as well as to **A** and **B** than to doxorubicin. Nonetheless, the cytotoxicity can be considered moderate [8]. In contrast to doxorubicin, which was about 1000 times less active in multidrug-resistant CEM/ADR5000 cells than in parental CCRF-CEM cells, compounds **A** and **B**, as well as the semi-synthetic compound **3**, showed minor cross-resistance in the otherwise highly drug-resistant CEM/ADR5000 cells. Regarding the structure-activity relationship, it clearly appeared that O-prenylation at position C4′ of **B** to afford **3** considerably reduced the cytotoxic activity. In addition to the O-prenylation, acetylation at C-2′ of **3** to afford **4** or at C5 of **1** to yield **2**, further reduced the antiproliferative activity. These data clearly suggest that semi-synthesis involving O-prenylation and acetylation of chalcones or chromones should be avoided in the search for potential anticancer drugs. Usually it has been found that C-prenylation of the flavonoid nucleus increases the cytotoxic activity [9]. Moreover, as an additional benefit, prenylated flavonoids are relatively non-toxic to non-cancer cells [9]. In our case, O-prenylation was accompanied by a decrease in bioactivity, indicating the importance of free phenolic groups. This conclusion is further supported by the observation that, after prenylation, acetylation of the remaining free phenolic groups further reduced the cytotoxicity.

**Table 2:** Cytotoxicity of compounds A, B, 1–4 towards sensitive and drug-resistant cancer cell lines and normal cells, as determined by the resazurin assay

Cell lines	Compounds (μM) and degrees of resistance (in brackets)						
	Flavonoid derivatives						Doxorubicin
	A	B^#^	1	2	3	4	
CCRF-CEM	7.85 ± 0.82	10.67 ± 0.73	(−)	(−)	18.07 ± 2.04	(−)	0.20 ± 0.06
CEM/ADR5000	15.03 ± 1.02	18.60 ± 2.60	(−)	(−)	19.70 ± 1.86	(−)	195.12 ± 14.30
Degree of resistance^a^	(1.91)	(1.74)			(1.09)		(975.60)

^a^The degree of resistance was determined as the ratio of IC_50_ value of the resistant/IC_50_ sensitive cell line; 5,7-dihydroxy-3-(1′-hydroxy-1′-phenyl-methyl)-6-methoxy-chroman-4-one (A), 2′,4′-dihydroxy-3′,6′-dimethoxychalcone (B); 5-hydroxy-6-methoxy-7-*O*-(3′,3′-dimethylprop-2′-enyl)chroman-4-one (1), 5-acetyl-6-methoxy-7-*O*-(3′,3′-dimethylprop-2′-enyl)chroman-4-one (2), 2′-hydroxy-3′,6′-dimethoxy-4′-*O*-(3″,3″-dimethylprop-2″-enyl)chalcone (3), 2′-acetyl-3′,6′-dimethoxy-4′-*O*-(3″,3″-dimethylprop-2″-enyl)chalcone (4).(−):>125 μM; # data previously reported [4]

## Conclusions

The objective of this study was to modify the structures of antitumor compounds **A** and **B** by O-prenylation and acetylation and to evaluate the structure-activity relationship (SAR). The results clearly suggest that O-prenylation and acetylation of chalcones, chromanones and possibly other flavonoids should be avoided in the search for potential anticancer drugs.

## Methods

The starting materials, 5,7-dihydroxy-3-(1′-hydroxy-1′-phenyl-methyl)-6-methoxy-chroman-4-one (**A**) and 2′,4′-dihydroxy-3′,6′-dimethoxychalcone (**B**), were isolated from the leaves of *Polygonum limbatum* as previously reported [3, 4]. The purity of semi-synthetic compounds **1**, **2**, **3**, and **4** was determined by analytical HPLC and was found to be > 98 %. Melting points were determined on a Büchi SMP-20 melting point apparatus and with a Reichert microscope and are uncorrected. IR spectra were recorded on a SHIMADZU FTIR-8400S spectrophotometer. EI-MS (ionization voltage 70 eV) and ESI-MS spectra were recorded on a Finnigan MAT double focusing spectrometer Model 8230. ^1^H NMR (300 MHz) and ^13^C NMR (75 MHz) spectra were recorded in CDCl_3_, DMSO-*d*_*6*_ and MeOD using a Varian Mercury Plus NMR spectrometer (7.05 T) and TMS as an internal reference. Silica gel 60 (70–230 mesh ASTM; Merck; Darmstadt, Germany) was used for column chromatography with step- gradients of *n*-hexane-EtOAc and EtOAc-MeOH as eluents. Precoated silica gel plates (Merck, Kieselgel 60 F_254_) were used for TLC. Spots were visualized at 254 and 365 nm, and by spraying with 50 % H_2_SO_4_ followed by heating at 100 °C.

### Plant material

*Polygonum limbatum* Meisn. was collected in Balatchi village in the Metap swampy area, near the city of Mbouda, Western Region of Cameroon in March 2010. The plant was identified at the Cameroon National Herbarium, Yaoundé, where a voucher specimen was deposited under the reference number 38852/HNC.

### Extraction and isolation

**Fig. 5:**
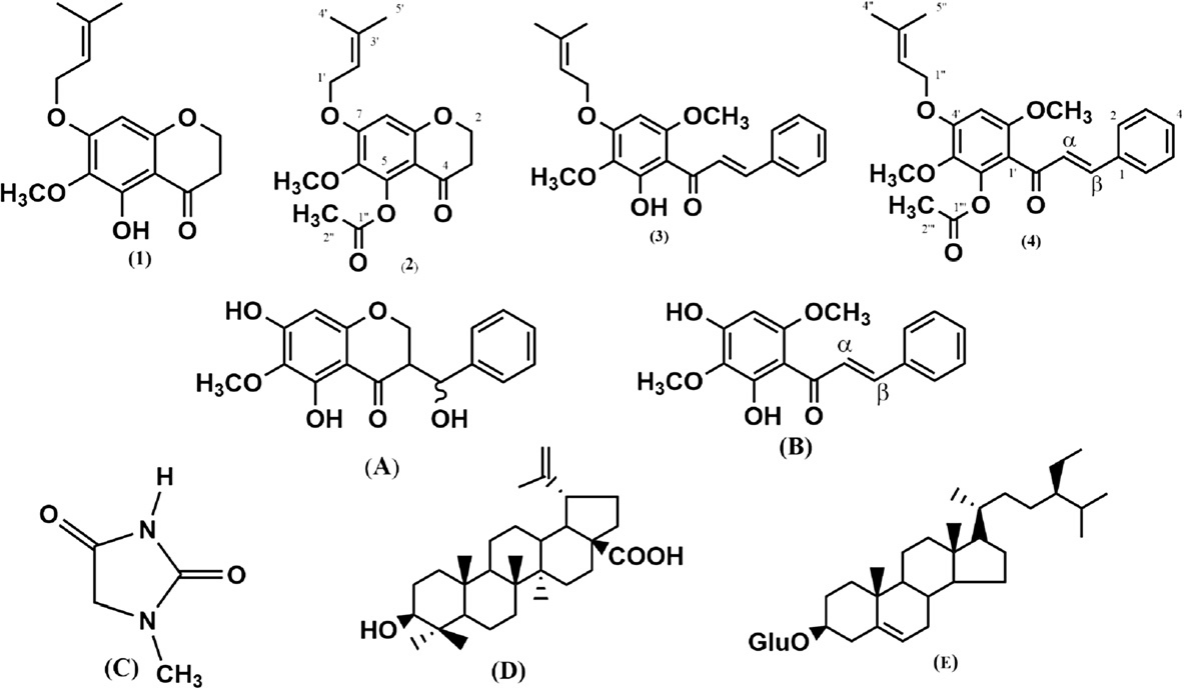
Chemical structures of compounds

We previously reported the isolation of 5,7-dihydroxy-3-(1′-hydroxy-1′-phenyl-methyl)-6-methoxy-chroman-4-one (**A**) and 2′,4′-dihydroxy-3′,6′-dimethoxychalcone (**B**) from the crude extract of *P. limbatum* [3]. A re-isolation of the compounds was performed following our previously described procedure [3] with some modifications that allowed the isolation of other flavonoids, 1-methylhydantoin (**C**) and terpenoids. A portion of the MeOH extract (7 g) was submitted to separation by column chromatography and HPLC, affording compounds **C**, **D** and **E** (Fig. 5).

### Semisynthetic compounds

#### O-Prenylation of 5,7-dihydroxy-3-(1′-hydroxy-1′-phenyl-methyl)-6-methoxy-chroman-4-one to metapchromone (1)

Compound **A** (10 mg, 30 × 10^−3^ mmol) was dissolved in 2 mL of acetone (0.1 M), and 1.7 mL of prenyl bromide and K_2_CO_3_ (3.4 mg, 38.6 × 10^−3^ mmol) were added successively. The mixture was stirred overnight at room temperature (Fig. 1). Distilled water (10 mL) was added to the mixture, which was stirred for 25 min. Extraction with CH_2_Cl_2_ and chromatographic purification on a silica gel column with mixtures of *n*-hexane-EtOAc gave a new derivative, 5-hydroxy-6-methoxy-7-*O*-(3′-methylbut-2′-enyl)chroman-4-one (**1**) (7.2 mg, 72 %), trivially named metapchromone.

##### 5-Hydroxy-6-methoxy-7-O-(3′-methylbut-2′-enyl)chroman-4-one (1)

Yellow powder; mp 75–77 °C; IR (KBr): ν_max_ = 2933, 1647, 1571, 1449, 1289, 1105, 808 cm^−1^; ESIMS:*m/z*579.30 [2M+Na]^+^; 301.09 [M+Na]^+^; 233.00.^1^H NMR (300 MHz, CDCl_3_) and ^13^C NMR (75 MHz, CDCl_3_): see Table 3.

**Table 3:** ^1^H NMR (300 MHz) and/or ^13^C NMR (75 MHz) data for compounds 1, 2, 3, 4 in CDCl_3_[δ(ppm), J(Hz)]

	1		2		3		4
Position	*δ*_*H*_	*δ*_*C*_	*δ*_*H*_	*δ*_*C*_	*δ*_*H*_	*δ*_*C*_	*δ*_*H*_
1	-	-	-	-	-	138.3	-
2	4.46(dd,6.54,12.86)	65.7	4.46(dd,6.54,12.86)	65.8	7.41(m)	127.4	7.41(m)
3	2.78(dd,6.55,12.87)	36.4	2.68(dd,5.13,14.07)	38.1	7.45(m)	128.2	7.45(m)
4	-	196.1	-	188.7	7.43(m)	128.7	7.43(m)
4a	-	103.3	-	107.7		-	
5	-	-	-	155.1	7.45(m)	128.5	7.45(m)
5-OH	11.91(s)	155.1	-	-		-	
6	-	128.9	-	128.9	7.42(m)	127.4	7.42(m)
6-OCH_3_	3.83(s)	60.6	377(s)	61.1	-	-	-
7	-	160.1	-	159.5	-	-	-
8	6.08(s)	92.2	6.38(s)	99.1	-	-	-
8a	-	158.6	-	158.5	-	-	-
1′	4.61(d,6.63)	66.6	4.61(d,6.63)	66.9	-	107.2	-
2′	5.48(brs)	118.7	5.50(brs)	118.3	-	158.2	-
2′-OH	-	-	-		13.93	-	-
3′	-	138.6	-	139.0	-	130.0	-
3′-OCH_3_	-	-	-		3.85	55.8	3.79(s)
4′	1.81(s)	25.6	1.83(s)	25.6	-	157.9	-
5′	1.76(s)	18.1	1.77(s)	18.2	6.04	88.5	6.41(s)
6′	-	-	-	-	-	159.4	-
6′-OCH_3_	-	-	-	-	3.93	60.5	3.79(s)
1″	-	-	-	169.3	4.71(d,6.57)	65.8	4.65(d,6.52)
2″	-	-	2.10(s)	20.8	5.52(brs)	119.2	5.52(brs)
3″	-	-	-	-	-	138.3	-
4″	-	-	-	-	1.83(s)	25.7	1.82(s)
5″	-	-	-	-	1.62(s)	18.2	1.78(s)
α	-	-	-	-	7.81(d,15.65)	127.4	7.01(d,16.06)
β	-	-	-	-	7.90(d, 15.65)	142.4	7.53(d,8.49)
C=O	-	-	-	-	13.93(s)	193.0	

#### Acetylation of 5-hydroxy-6-methoxy-7-*O*-(3′-methylbut-2′-enyl)chroman-4-one (1) to sargisin (2)

Compound **1** (4 mg, 10 × 10^−3^ mmol) was dissolved in pyridine (0.5 mL); Ac_2_O (8 mL) and 4-dimethylaminopyridine (DMAP) (1 mg) were added, and the mixture was stirred at room temperature for 24 h. H_2_O (8 mL) was then added to the mixture, which was stirred for 30 min (Fig. 1). Extraction with CH_2_Cl_2_ and chromatographic purification on a Sephadex LH-20 gel column with CH_2_Cl_2_/MeOH (6:4) as isocratic eluent gave a new derivative, 5-acetoxy-6-methoxy-7-*O*-(3′-methylbut-2′-enyl)chroman-4-one (**2**) (3.7 mg, 92.5 %), which was crystallized from *n*-hexane-EtOAc (7:3) and trivially named sargisin.

##### 5-Acetoxy-6-methoxy-7-O-(3′-methylbut-2′-enyl)chroman-4-one (2)

Brownish powder, mp 87–89 °C, ESIMS:*m/z* 663.06 [2M+Na]^+^, 603.33 [2M-AcOH+Na]^+^, 543.40 [2M-2AcOH+Na]^+^, 343.34 ([M+Na]^+^; ^1^H NMR (300 MHz, CDCl_3_) and^13^C NMR (75 MHz, CDCl_3_) see Table 3.

#### O-Prenylation of 2′,4′-dihydroxy-3′,6′-dimethoxychalcone (B) to limbachalcone A (3)

**Fig. 6:**
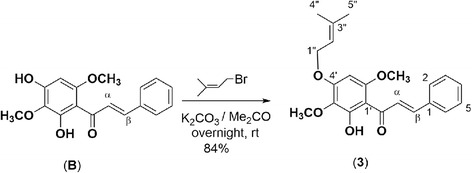
Semi-synthesis of compound **3** from 2′,4′-dihydroxy-3′,6′-dimethoxychalcone (**B**)

Compound **B** (10 mg, 30 × 10^−3^ mmol) was dissolved in 3 mL of acetone (0.1 M); prenyl bromide (2 mL) and K_2_CO_3_ (3 mg) were added successively. The mixture was heated at 40 °C for 3 h (Fig. 6). Distilled H_2_O (10 mL) was then added to the mixture, which was stirred for 40 min. Extraction with CH_2_Cl_2_ (3 × 10 mL)_,_dryingover Na_2_SO_4_, and chromatographic purification on a silica gel column with mixtures of *n*-hexane-EtOAc yielded, after crystallization, 2′-hydroxy-3′,6′-dimethoxy-4′-*O*-(3″-methylbut-2″-enyl)chalcone (**3**) (8.4 mg, 84 %), trivially named limbachalcone A.

##### 2′-Hydroxy-3′,6′-dimethoxy-4′-O-(3″-methylbut-2″-enyl)chalcone (3)

Yellowish powder; mp 98–100 °C;IR (KBr): ν_max_ = 2931, 1629, 1560, 1334, 1119, 792 cm^−1^; ^1^H (300 MHz, CDCl_3_) and ^13^C NMR (75 MHz, CDCl_3_); ESIMS: *m/z*1126.92 [3M+Na]^+^, 759.83 [2M+Na]^+^, 391.25 [M+Na]^+^.

#### Acetylation of 2′-hydroxy-3′,6′-dimethoxy-4′-*O*-(3″-methylbut-2″-enyl)chalcone to tsedengchromone (4)

**Fig. 7:**
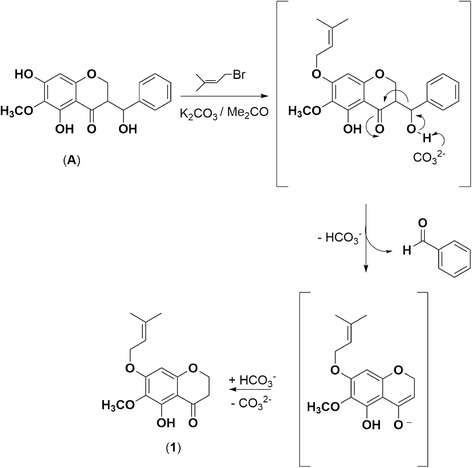
Semi-synthesis of compound **4** from 2′-hydroxy-3′,6′-dimethoxy-4′-*O*-(3″-methylbut-2″-enyl)chalcone (**3**)

Compound **1** (3.2 mg, 10 × 10^−3^ mmol) was dissolved in pyridine (0.4 mL); Ac_2_O (5 mL) and DMAP (1 mg) were added, and the mixture was stirred at room temperature for 24 h (Fig. 7). Distilled H_2_O (5 mL) was then added to the mixture, which was stirred for 30 min. Extraction with CH_2_Cl_2_ and chromatographic purification on a Sephadex LH-20 gel column with CH_2_Cl_2_/MeOH (1:1) as isocratic eluent, yielded 2′-acetoxy-3′,6′-dimethoxy-4′-*O*-(3″-methylbut-2″-enyl)chalcone (**4**) (2.8 mg, 87.5 %), which was crystallized from *n*-hexane-EtOAc (6:4) and trivially named tsedengchromone.

##### 2′-Acetoxy-3′,6′-dimethoxy-4′-O-[3″-methylbut-2″-enyl]chalcone (4)

Brownish powder; mp 102-104 °C; ESIMS: *m/z* 843.13 [2M+Na]^+^, 433.42 [M+Na]^+^; ^1^H NMR (75 MHz, CDCl_3_) see Table 3.

##### 1-Methylhydantoin (3-methyl-2,4-imidazolidinedione) (C)

Brownish powder; mp 155-157 °C, ^1^H NMR (75 MHz, MeOH-d_4_); *δ* = 3.95 (3H, s, CH_3_); 2.90 (2H, s,CH_2_); ^13^C NMR (75 MHz, MeOH-d_4_); see Table 2; EIMS *m/z* (rel. int.): 114 (M^+^, 35), 86 (6), 42 (72).

### Cytotoxicity assay

The resazurin reduction assay [10] was performed to assess the cytotoxicity of compounds and doxorubicin was used as a control drug towards the parental, drug-sensitive CCRF-CEM leukaemia cell line and its multidrug-resistant, P-glycoprotein-over-expressing subline, CEM/ADR5000. The assay is based on the reduction of the indicator dye, resazurin, to the highly fluorescent resorufin by viable cells. Non-viable cells rapidly lose their metabolic capacity to reduce resazurin and, thus, do not produce fluorescent signals anymore. Briefly, aliquots of 2 × 10^4^ cells per well were seeded in 96-well-plates in a total volume of 100 μL. The studied compound was immediately added at varying concentrations to an additional 100 μL of culture medium to obtain a total volume of 200 μL/well. After 72 h, resazurin (Sigma-Aldrich, Schnelldorf, Germany) (20 μL, 0.01 % w/v) in distilled H_2_O was added to each well and the plates were incubated at 37 °C for 4 h. Fluorescence was measured on an Infinite M2000 ProTM plate reader (Tecan, Crailsheim, Germany) using an excitation wavelength of 544 nm and an emission wavelength of 590 nm. A preliminary assay was done with all samples on leukaemia CCRF-CEM cells at 125 μM. Each assay was done at least twice with six replicates each. The viability was evaluated by comparison with untreated cells. IC_50_ values represent the compound concentrations required to inhibit 50 % of cell proliferation and were calculated from a calibration curve by linear regression with the aid of Microsoft Excel [11].
